# Nano-Based Drug Delivery Systems of Potent MmpL3 Inhibitors for Tuberculosis Treatment

**DOI:** 10.3390/pharmaceutics14030610

**Published:** 2022-03-10

**Authors:** Patrizia Nadia Hanieh, Sara Consalvi, Jacopo Forte, Gianluigi Cabiddu, Alessandro De Logu, Giovanna Poce, Federica Rinaldi, Mariangela Biava, Maria Carafa, Carlotta Marianecci

**Affiliations:** 1 Dipartimento di Chimica e Tecnologie del Farmaco, Sapienza Universitaà di Roma, Piazzale Aldo Moro 5, 00185 Rome, Italy; patrizianadia.hanieh@uniroma1.it (P.N.H.); sara.consalvi@uniroma1.it (S.C.); jacopo.forte@uniroma1.it (J.F.); giovanna.poce@uniroma1.it (G.P.); mariangela.biava@uniroma1.it (M.B.); maria.carafa@uniroma1.it (M.C.); carlotta.marianecci@uniroma1.it (C.M.); 2 Department of Life and Environmental Sciences, Laboratory of Microbiology, University of Cagliari, Cittadella Universitaria di Monserrato, 09042 Monserrato, Italy; cabgianluigi@gmail.com (G.C.); adelogu@unica.it (A.D.L.)

**Keywords:** nanocarriers, niosomes, nanoemulsions, tuberculosis

## Abstract

Tuberculosis remains one of the world’s deadliest infectious diseases, accounting for nearly 1.3 million deaths every year. Tuberculosis treatment is challenging because of the toxicity, decreased bioavailability at the target site of the conventional drugs and, most importantly, low adherence of patients; this leads to drug resistance. Here, we describe the development of suitable nanocarriers with specific physicochemical properties to efficiently deliver two potent antimycobacterial compounds. We prepared nanoemulsions and niosomes formulations and loaded them with two different MmpL3 inhibitors previously identified (NEs + BM635 and NIs + BM859). NEs + BM635 and NIs + BM859 were deeply characterized for their physicochemical properties and anti-mycobacterial activity. NEs + BM635 and NIs + BM859 showed good hydrodynamic diameter, ζ-Potential, PDI, drug-entrapment efficiency, polarity, and microviscosity and stability. Even though both formulations proved to perform well, only NIs + BM859 showed potent antimycobacterial activity against *M. tuberculosis* (MIC = 0.6 µM) compared to that of the free compound. This is most probably caused by the fact that BM635, being highly hydrophobic, encounters maximum hindrance in diffusion, whereas BM859, characterized by high solubility in aqueous medium (152 µM), diffuses more easily. The niosomal formulation described in this work may be a useful therapeutic tool for tuberculosis treatment, and further studies will follow to characterize the in vivo behavior of the formulation.

## 1. Introduction

Infectious diseases remain one of the most life-threatening challenges in the modern world, regardless of advanced technology and innovation. Prior to the COVID-19 pandemic, tuberculosis (TB) was the leading cause of deaths worldwide from a single infectious agent, accounting for 1.3 million deaths in 2020 [1]. Even though the combination of timely diagnosis and treatment with first-line drugs for six months can cure people who develop drug-susceptible TB and prevent the transmission of infection, cure success rates are very low. Indeed, inappropriate use of anti-TB medicines, poor quality drugs, and poor patience compliance can cause the emergence of drug-resistant strains. Multi-drug resistant TB (MDR-TB) is caused by *M. tuberculosis* (*Mtb*) strains resistant to isoniazid (INH) and rifampicin (RIF), whereas extensively drug-resistant TB (XDR-TB) is caused by *Mtb* strains resistant to INH and RIF, in addition to any fluoroquinolone and at least bedaquiline or linezolid, if not both. The World Health Organization (WHO) estimates that the success rate of treatment of MDR/XDR TB patients was 59% in 2018 [1]. Therefore, there is an urgent need to develop a faster-acting and simplified TB regimen containing new drugs without pre-existing resistance.

**Figure 1:**
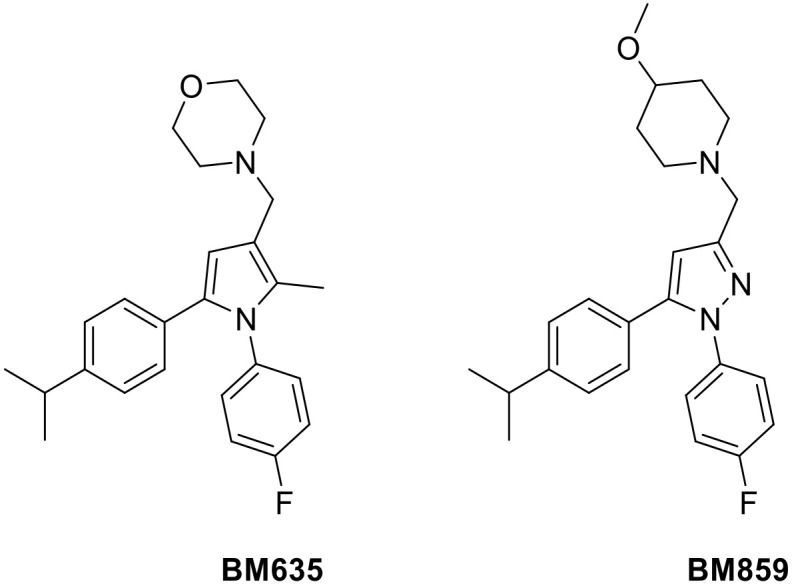
Chemical structures of BM635 and BM859.

In this scenario, we have developed a potent class of antimycobacterial compounds acting as inhibitors of the essential mycobacterial membrane protein large 3 (MmpL3). Among this class of compounds, the 1,5-diarylpyrrole BM635 and the 1,5-diarylpyrazole BM859 (Figure 1) showed outstanding in vitro activity against *Mtb* (minimum inhibitory concentration (MIC) = 0.12 µM and 0.3 µM, respectively) as well as good in vivo efficacy in a murine model of TB infection. The main drawback of these potent compounds was the high lipophilicity (8.1 Chrom log D_pH 7.4_ and 7.31 Chrom log D_pH 7.4_, respectively) [2,3]. 

Nanocarriers for drug delivery represent an interesting and efficient strategy to overcome many pharmacological conventional therapies’ limits such as: poor drug bioavailability, drug in vivo degradation/inactivation, and side effects (due to high administration doses). Niosomes (NIs) are vesicular drug delivery systems composed by surfactants characterized by the same liposome structures: an inner aqueous core entrapped within a lipophilic bilayer. Due to this internal structure, vesicular nanocarriers are able to entrap lipophilic drugs and hydrophilic ones.

It is possible to prepare both vesicular systems as unilamellar or multilamellar structures by employing the same preparation methods but modifying some experimental conditions/parameters, and nanocarriers with specific physical–chemical characteristics can be obtained. Compared to liposomes, niosomes are characterized by great advantages related to their low costs and their high versatility. In fact, it is possible to derivatize the different surfactants with molecules that confer to the nanocarriers’ stimuli-responsive behavior.

An ever-increasing interest has been exhibited on the application of NIs in the field of pharmaceutics, cosmetics, and the food industry, leading to the publication of more than 1325 research articles, about 200 patents and 6 clinical trials from 1980 (from Scopus.com accessed on 28 January 2022). In particular, Tween 20 is a non-ionic surfactant, the stability and relative non-toxicity of which allows it to be used as an excipient for oral administration [4,5].

Nanoemulsions (NEs) are a different type of nanocarrier with respect to NIs that represent promising drug delivery systems for antimycobacterial compounds delivery for tuberculosis treatment. Both systems are amphiphilic-based nanocarriers but with different internal structure and molecule organization, which affect their loading and delivery properties. NEs are composed of surfactant and oils, they are translucent systems, kinetically stable, and they show good entrapment efficiency. Oil in water (O/W) Nes, due to the presence of oil droplets, can load and delivery lipophilic drugs. Surfactants, Nes’ fundamental components, reduce the interfacial tension resulting in the formation of stable NEs as well as confer good physical–chemical characteristics. The choice of oil and surfactant is a crucial point in designing and preparing specific and efficient NEs. Indeed, functional NEs oil properties can have a synergic effect with the entrapped drug in order to potentiate the overall system activity; moreover, an efficient NEs is characterized by a low surfactant concentration useful to assure drug permeation but capable to prevent side or toxic effects. 

The preferred pathway for administering anti-TB drugs is the oral route because this way is painless, non-invasive, and consequently invokes a great degree of patients’ compliance and this, in turn, means a good degree of adherence to a therapeutic regimen [6].

BM635 and BM859 have been chosen for this study because they proved to be the most active compounds of the MmpL3 inhibitors series previously developed. Both drugs were characterized by several issues, such as high lipophilicity as well as low in vivo bioavailability. 

Indeed, to enhance the BM635 and BM859 oral bioavailability, protect these drugs from inactivation, and possibly reduce their administered doses, a possible and efficient strategy could be represented by drug delivery systems. Surfactants are frequently encountered in classical and enabling formulations (e.g., surfactants-based formulations) and they could contribute to drug solubility enhancement, as well as after application. In particular, the use of nanocarriers to improve the oral bioavailability of specific drugs or therapeutic peptides and proteins is an area of extensive research. However, the oral delivery of nanocarriers is challenging, due to the inherently unfavorable physio-chemical properties of the gastrointestinal tract; a different pH range or the presence of digestive enzymes in the gastrointestinal tract could affect free-drug and nanocarriers’ stability. These several physiological conditions could represent obstacles to the bioavailability and efficacy of the loaded active molecules. Therefore, to obtain an efficient drug delivery system suitable for the oral administration route, the designing, preparation, and characterization steps are crucial. In this work, the authors selected and proposed NEs/NIs by Tween 20 as gastro-resistant nanovectors for the oral administration of antimycobacterial compounds [7,8,9].

**Figure 2:**
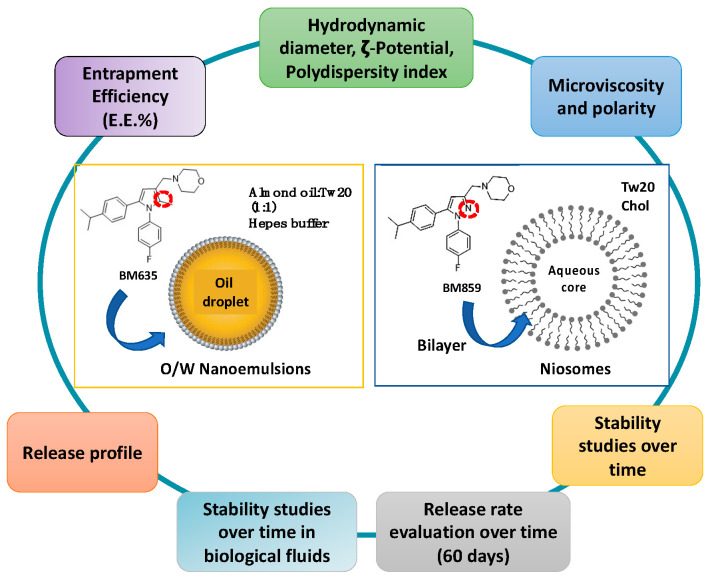
Nanocarriers’ physiochemical properties that were investigated.

Preliminary studies were carried out to select the most appropriate nanocarrier for both compounds and, even if NIs vesicles should be able to entrap both lipophilic and hydrophilic drugs [10,11,12], the more lipophilic BM635 could not be entrapped into NIs [data not showed], and NEs were selected as the best formulation for BM635, whereas BM859 could be loaded into NIs. We report herein the preparation and physical-chemical characterization of the two different formulations (summarized in Figure 2). In particular, together with nanocarrier morphological characteristics, their stability in different biological media such as Simulated Gastric Fluid (SGF), Simulated Intestinal Fluid (SIF) and Human Serum (HS) was evaluated. Moreover, the in vitro activity against *Mtb* of NEs and NIs loaded with BM635 and BM859 was evaluated and compared with free drug activity.

## 2. Materials and Methods

### 2.1. Materials

Tween 20, Almond Oil, Cholesterol, Hepes salt (N-(2-hydroxyethyl) piperazine-*N*-(2-ethanesulphonic acid)), pyrene, ethanol, trypsin, pepsin, sodium chloride and human serum were purchased by Sigma-Aldrich (St. Louis, MO, USA). BM635 and BM859 were prepared following a procedure reported in the Figures S1 and S2 from the Supplementary Materials.

### 2.2. Ternary Diagram Construction and Empty and Loaded NEs Preparation

The ternary phase diagram of Almond Oil NEs was developed. The mixtures were prepared by combining the appropriate amounts of surfactant, oil phase, and aqueous phase (Hepes buffer, pH 7.4) in different weight ratios (Table S1) in a test tube and were vortexed vigorously for 10 s to ensure thorough mixing. Visual inspection was made after each sample preparation. The best formulation of NEs prepared and characterized with BM635 was selected by the homogenous phase (blue zone, Table S1, sample 52) of ternary diagram. To allow the NEs to form, the selected compounds (Table 1) together with BM635 (for loaded NEs) were mixed using an UtraTurrax (IKA^®^ T18, Staufen, Germany) for 1 min at 5000 rpm, and then, the obtained microscale droplets were sonicated for 20 min at 50 °C using a tapered microtip operating at 20 kHz at an amplitude of 18% (Vibracell-VCX 400, Sonics, Taunton, MA, USA) to obtain the NEs. At this stage, all formulations could be sterilized using cellulose filters (0.22 μm) in accordance with Ph. Eur.

### 2.3. Preparation of NIs and Drug-Loaded NIs

NIs and drug-loaded NIs were prepared by Thin Layer Evaporation technique as previously reported [13]. Sample composition is reported in Table 1. Lipophilic components and BM859 were solubilized in an organic mixture (CH_3_Cl:CH_3_OH, 3:1 *v*/*v*) that was evaporated for one hour using a Rotavapor^®^ R-210 (Büchi-Italia S.r.l., Assago, Italy) and an oil pump under vacuum to obtain a thin lipid film. The suspension was then sonicated for 5 min at 60 °C with 16% of amplitude using a microprobe operating at 20 kHz (Vibracell-VCX 400, Sonics, Taunton, MA, USA). Finally, centrifugation (MPW-260R) was carried out to purify the vesicle suspension from unloaded components remaining in the precipitate at 10,000 RPM for 10 min at 15 °C.

### 2.4. Dynamic Light Scattering and ζ-Potential Measurements

The obtained samples were characterized in terms of hydrodynamic diameter, ζ-Potential, and size distribution (polydispersity index, PDI) using a Malvern NanoZetaSizer apparatus (Malvern Instruments, Worcestershire, UK) equipped with a 5 mW HeNe laser, λ = 632.8 nm. All samples were diluted with Hepes buffer just before measurement to avoid multiple scattering phenomena.

Contin algorithm was used for the analysis of the intensity autocorrelation function at 90° as scattering angle. The hydrodynamic diameter and the polydispersity index (PDI) were obtained after data analysis by the cumulant method [14].

The calculated mean hydrodynamic radius corresponds to the intensity weighted average [15]. Electrophoretic mobility (u) of the vesicles was measured by laser Doppler anemometry using the Malvern Zetasizer Nano ZS90 apparatus.

The Smoluchowski relation ζ = uη/ð, where η is the viscosity and ð the permittivity of the solvent phase, was used to obtain the ζ-potential value by electrophoretic mobility measurement [16].

### 2.5. Fluorometric Measurements

Pyrene is a fluorescent probe employed to obtain information about oil droplet and niosomes bilayer features. The probe (4 mM) was added inside both nanocarriers together with lipophilic compounds maintaining the same preparation method previously described for NIs and NEs preparation. The fluorescent signals emitted by pyrene loaded inside both nanocarriers were collected; the emission spectrum (λ = 350–550 nm) and Ex = 330 nm were evaluated using a luminescence spectrometer (LS5013, PerkinElmer, Waltham, MA, USA) [17]. The probe spectrum is characterized by five emission bands (I1–I5) as monomer and one as excimer (IE), and their fluorescence intensities depend on the lateral distribution and mobility of the probe into the oil droplet or into the niosomes bilayer. By the I1/I3 ratio (first and third vibration bands respectively), it is possible to obtain information about the polarity of the probe environment, whereas the ratio between IE and I3 provides information about viscosity [18].

### 2.6. Drug-Entrapment Efficiency (E.E.%)

Entrapment efficiency (EE) of drugs in NEs and NIs vesicles was evaluated by UV-vis spectrometer (Lambda 25, PerkinElmer, Waltham, MA, USA). In particular, the active compound concentration was calculated using the calibration curves previously determined. Both samples were diluted in Ethanol:Hepes 70:30 *v:v* and absorbance of drugs at λ = 285 nm for BM635 and at λ = 252 nm for BM859 was measured.

*E.E.%* was calculated as (1):

(1) $$E.E.\;\left(\%\right)=\frac{Entrapped\;drug\;\left(\mathrm{mg}\right)}{Total\;drug\;used\;\left(\mathrm{mg}\right)}\times 100$$

### 2.7. Physicochemical Stability

The empty and drug-loaded NEs and NIs formulations were stored at 4 °C and room temperature for a period of 30 days, and stability studies were carried out by DLS (Malvern Instruments, Worcestershire, UK) and UV-VIS (Lambda 25, PerkinElmer, Waltham, MA, USA). Samples from each batch of empty nanosystems and drug-loaded NEs and NIs were withdrawn at fixed time intervals (1, 7, 14, 21 and 30 days), and the mean of hydrodynamic diameter and PDI, ζ-potential were determined as previously described. 

The stability of free drugs and drug-entrapment in niosomes and nanoemulsions was also controlled at two different temperatures, 4 °C and room temperature, and after 1, 7, 14, 21 and 30 days were evaluated by UV-vis spectrometer (Lambda 25, PerkinElmer, Waltham, MA, USA).

### 2.8. NEs and NIs Biological Stability

The biological stability of NEs and NIs in the presence of SGF, SIF, and HS were evaluated. In particular, a mixture of NEs and Nis, and 45% of the fluid was prepared and placed at 37 °C. By DLS (Malvern Instruments, Worcestershire, UK), the mixture was analyzed at different time intervals (0, 0.5, 1, 2 and 3 h) to evaluate the particle size and ζ-potential. 

### 2.9. NEs and NIs Stability in Culture Medium

As a preliminary biological evaluation, the in vitro stability of NEs and NIs in the presence of culture medium Middlebrook 7H9 medium (Difco, Franklin Lakes, NJ, USA) used for MIC determination was carried out. Samples were diluted in culture medium to obtain a final concentration of 45%. The average size, polydispersity index, and ζ-potential were evaluated by means of DLS (Malvern Instruments, Worcestershire, UK), maintaining samples at 37 °C and performing measurements at different time points (for 7 days).

### 2.10. In Vitro Release Studies

In vitro release experiments were performed to test drug release from NEs and NIs. Experiments were carried out using dialysis tubes (molecular weight cut-off: 8000 MW by Spectra/Por^®^, Rancho Dominguez, CA, USA) at 37 °C in a release medium Ethanol:Hepes 70:30 *v/v*, which was gently magnetically stirred during the experiment.

Drug concentration in the release medium was measured using the UV spectrophotometer (Lambda 25, PerkinElmer, Waltham, MA, USA). The experiments were carried out over 24 h; 1 mL of acceptor compartment was withdrawn to evaluate the quantity of drugs released by both nanocarriers. The measurement was carried out at selected interval times: from 1 h to 8 h hourly and after at the 24 h mark. Each aliquot that was withdrawn to perform UV analyses was then reinserted back into the acceptor compartment. The experiment was repeated three independent times and the errors represent the standard deviation.

### 2.11. MIC Determination

The activity of Nes + BM635 and Nis + BM859 was evaluated against *Mtb* H37Rv ATCC 27294 by the resazurin microtiter assay (REMA) plate method. Formulations were diluted in Middlebrook 7H9 medium with 10% ADC (bovine Albumin Fraction V 0.5%, Dextrose 0.2%, Catalase 0.003%) enrichment to obtain a concentration of 256 μg/L. Serial two-fold dilutions of each formulation were prepared directly in sterile, flat-bottom, 96-well plates containing 100 μL/well of 7H9 medium. Each well received the same volume of *Mtb* H37Rv inoculum obtained from fresh cultures in 7H9, diluted to a McFarland standard of 1.0, and then further diluted 1:20 in the same medium. Final concentrations of NEs + BM635 and NIs + BM859 ranged between 0.00025 and 128 μg/L, and the final inoculum of *Mtb* H37Rv was 2.5 × 10^5^ cells/mL. Serial dilutions of empty NEs and NIs were also evaluated to verify any potential interaction with the growth of mycobacteria, as well as of free compounds. A growth control and a sterile control were also included. Plates were incubated at 37 °C for 7 days in 5% CO_2_. Next, 30 μL of freshly prepared filter-sterilized 0.01% resazurin solution in H_2_O was added to each well. Plates were incubated for an additional 24 h at 37 °C. A color change from blue to pink indicated mycobacterial growth. The MIC was determined as the lowest concentration that prevented the color change. All experiments were performed in triplicate. Isoniazid was employed as reference drug in all assays.

### 2.12. Statistical Analysis

The results of physicochemical and biological characterization are expressed as the mean of three independent experiments ± standard deviation (SD). These statistical analyses were performed using Student’s *t*-test and a *p*-value lower than 0.05 was considered statistically significant.

## 3. Results and Discussion

### 3.1. NEs and NIs Characterization

**Figure 3:**
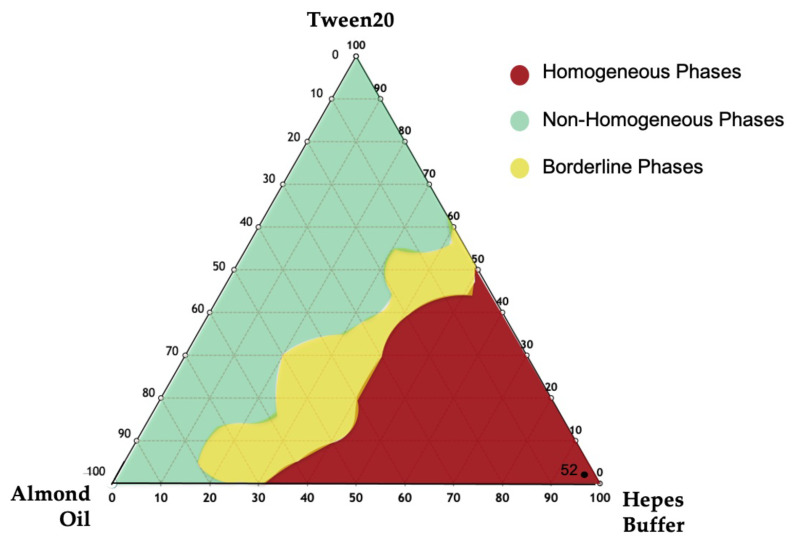
Ternary phase diagrams among Almond oil, Tween 20, and Hepes buffer. The observed phases were the homogeneous phase (red area), the non-homogenous phase (green area), and the borderline phase region (yellow area). NEs in the red region were chosen.

To select the appropriate NEs in terms of hydrodynamic diameter, ζ-potential, and PDI, the ternary phase diagram of almond oil was developed. At first, different formulations were evaluated by visual inspection to delimit the three different zones in the diagram (Figure 3), which included homogenous and non-homogenous ones. In fact, only employing specific ratios of oil, surfactant and water phase (HEPES buffer) at specific concentration, it is possible to obtain a homogeneous dispersion without separation phenomena. The red region represents the homogenous region, and the green zone is the non-homogenous one. In addition, the sonication performed on the samples in the non-homogenous zone led to formation of monophasic dispersion represented by the yellow zone (or borderline zone) [19,20,21].

To optimize the monophasic emulsion in the red region as a suitable drug delivery system, all samples were sonicated (at the conditions described in Materials and Methods). A better formulation in terms of hydrodynamic diameter, ζ-potential, and PDI was selected (Table S1 sample 52, Supplementary Materials) and the same qualitative–quantitative surfactant composition was used to prepare NIs samples.

The total component amounts employed for NEs/NIs preparation are reported in Table 1.

To obtain an efficient entrapment efficiency value, taking into account the different water solubility and lipophilicity of BM859 and BM635, two different nanocarriers were selected. In particular, BM635 and BM635 were respectively loaded into NEs and NIs formulations.

The physical–chemical properties of NEs and Nis either empty or loaded with BM635 and BM859 are reported in Table 2. NEs and NIs samples were characterized in terms of hydrodynamic diameter, ζ-Potential, PDI, drug-entrapment efficiency, polarity and microviscosity (Table 2). First, it can be observed that the entrapment efficiency (E.E.%) of the drugs, evaluated by UV analysis, is very high for both formulations (Nes: 95.6%/Nis: 81.6%), and the size of the empty and loaded NEs and NIs remains in the same range. In fact, for loaded NIs and NEs, only a slight and not significant increase in the hydrodynamic diameter was recorded when drugs were loaded.

Moreover, the PDI values for both empty and loaded nanocarriers is compatible with a monodisperse population. The ζ-potential values are negative (NEs: −25.9/NIs: −19.4) and they are able to assure a good formulation stability, enhancing repulsion effects that prevent aggregation or precipitation phenomena [22,23].

All formulations are characterized by the same parameters in terms of hydrodynamic diameter, ζ-potential, and PDI, and as such, it is possible to compare the in vitro results of the nanocarriers. Indeed, it is well known that nanocarrier features affect cell internalization and have a direct impact on their efficacy or toxicity [24]. 

In order to characterize the nature of the NEs lipophilic droplet and NIs bilayer, the microviscosity and polarity parameters were evaluated. Pyrene-loaded nanocarriers were prepared following the procedure previously described, and the fluorescence spectrum allowed the calculation of the values of polarity and microviscosity reported in Table 2. The polarity values were quite similar for empty and loaded nanocarriers, but microviscosity values increased with BM635/BM859 inclusion. The increase in microviscosity (Table 2) could be influenced by BM635/BM859 localization in the oil phase/bilayer of NEs and NIs respectively, in agreement with the compounds’ nature. It is likely that the slight increase in the microviscosity value seen for NIs/NIs + BM859 (0.67–0.82) with respect to NEs/NEs + BM635 (1.87–2.15) was caused by the one order of magnitude lower lipophilicity of BM859 compared to BM635 (8.1 versus 7.3).

#### Physicochemical Stability

**Figure 4:**
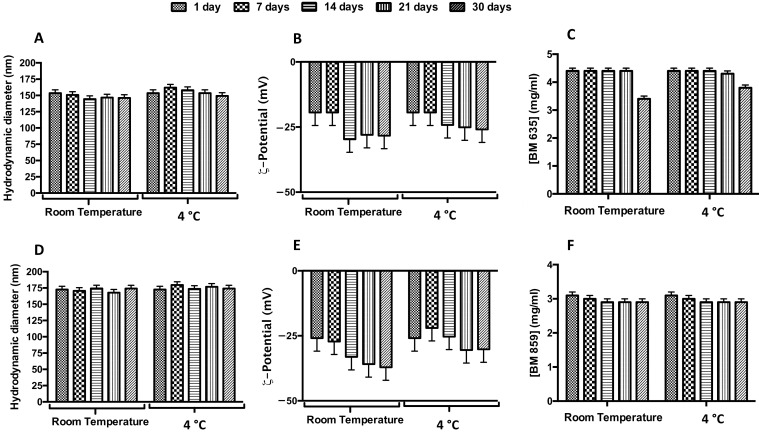
Physical-chemical stability over time of loaded nanocarriers. Effect of storage temperature (room temperature, RT; and 4 °C) on hydrodynamic diameter (panels (**A**,**D**)), ζ-potential (panels (**B**,**E**)) and concentration (panels (**C**,**F**)) for BM635-loaded NEs and BM859-loaded Nis. Data were obtained as the mean of three independent experiments.

To study the stability over time of NEs and NIs formulations, the samples were stored at both room temperature and 4 °C for a period of 30 days (Figure 4, panel B and E). It is possible to observe that during the storage, zeta potential increased, in modulus, with the accommodation and stabilization of the system, but these parameters ensure nanocarriers repulsion and their stability over time [23,25]. So, it is possible to affirm that NEs and NIs were stable under these experimental conditions due to the negative ζ-potential values able to prevent aggregation or precipitation phenomena. 

In order to evaluate drugs’ stability in terms of decomposition/degradation inside the nanocarriers, NEs + BM635 and NIs + BM859 were analyzed by UV analysis. The UV spectra were recorded immediately after the samples’ preparation and after 7, 14, 21 and 30 days (and up to 60 days, Figure S3, Supplementary Materials) at two different temperatures (room temperature and 4 °C). Drug concentration values are reported in Figure 4, panel C and F. BM635 and BM859 concentrations remained constant during the time interval analyzed when loaded into NEs/NIs. We may conclude that drug inclusion in nanocarriers leads to significant benefits in terms of drug-degradation protection and increased water solubility (by NEs/NIs inclusion). Indeed, at this concentration, the free drugs cannot be dissolved in water or HEPES buffer.

### 3.2. Nes and Nis Biological Stability

**Figure 5:**
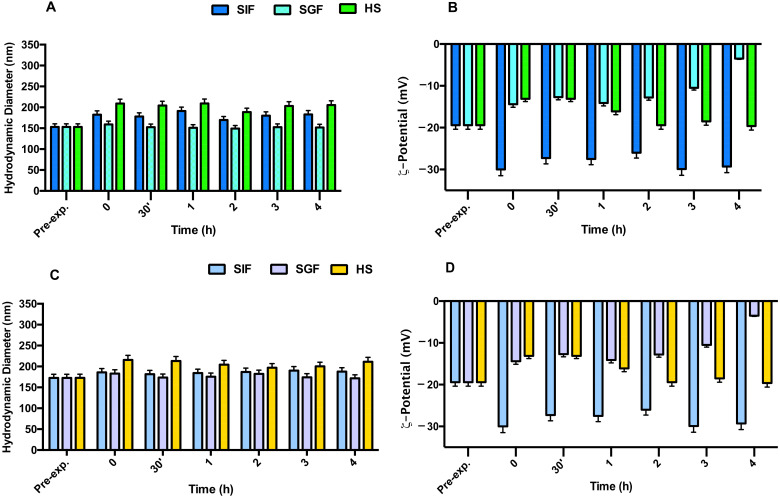
Stability studies in the presence of simulated intestinal fluid (SIF), simulated gastric fluid (SGF), and Human serum (HS) following variation in terms of hydrodynamic diameter and ζ-potential: panel (**A**,**B**) for NEs + BM635 and panel (**C**,**D**) NIs + BM859. Reported data represent the mean of three experiments ± SD.

To obtain an efficient drug delivery system for oral administration, loaded Nes and Nis should be resistant to the harsh gastrointestinal conditions. To test that, the stability of both NEs + BM635 and NIs + BM859 was evaluated in simulated gastrointestinal conditions. The physical stability of empty (Figure S4, Supplementary Materials) and loaded (Figure 5) NEs and NIs was assessed in SGF (pH 2) and in SIF (pH 7) following the previously described experimental conditions. The obtained results (Figure 5) demonstrate that these media did not affect the Nes/Nis integrity after 3 h of incubation; in fact, DLS analyses revealed that both hydrodynamic diameter and ζ-potential values of all formulations remained constant at the experimental conditions. For both samples, the ζ-potential in SIF decreased notably (values around −30 mV) immediately after incubation but remained constant during the time interval analyzed, assuring a good repulsion between dispersed particles. We may conclude that the different pH values and compositions of both simulated fluids have no effects on the nanocarriers’ integrity.

Similar results were obtained when NEs/NIs were incubated in HS (Figure 5). Indeed, even if it is possible to observe a slight increase in nanocarrier dimensions, due to the surface absorbance of serum protein, the Nis and Nes’ integrity was maintained during the experiment. 

### 3.3. Release Studies

**Figure 6:**
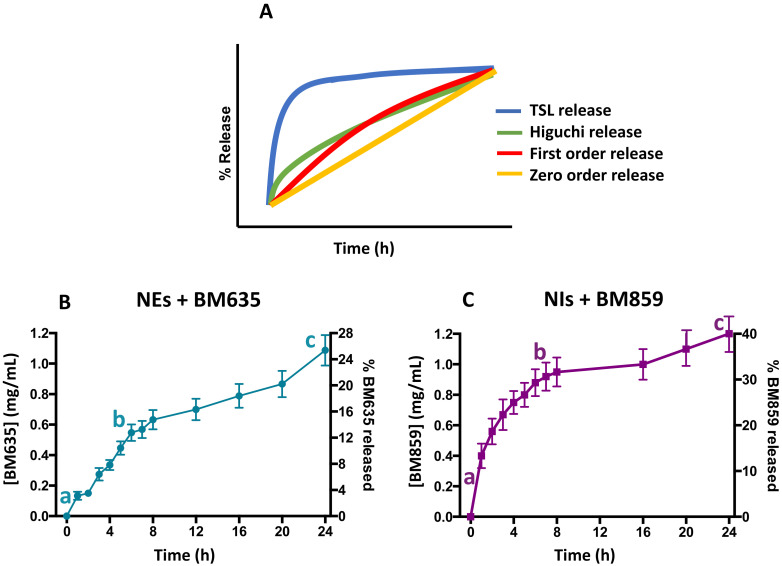
Kinetic model release profile (panel (**A**)) and drug release studies from nanoemulsions (panel (**B**)) and niosomes (panel (**C**)) in which faster release initially (a–b), followed by a relatively slow release (b–c) is shown.

In order to evaluate the NEs-NIs’ capability to release the entrapped drugs, release studies were performed. The aim of these studies was to understand how the different nanocarriers could influence the release of the entrapped drugs. In particular, Figure 6 shows the concentration versus time profiles of both active compounds released by the formulations. Both nanocarriers show high initial release, followed by a decreasing release rate. From Figure 6, it is possible to hypothesize the release kinetic model in comparison with literature data. In this case, the release curves of both formulations could be described as a bimodal release ref. More specifically, there was a faster release initially (a–b), followed by a relatively slow release (b–c), thus forming a “Γ” shape-like curve [26]. It is possible to observe that the total amount of BM635 released from NEs is around 30%, whereas 40% of BM859 is released from Nis. The slightly different release could be due to the different microviscosity values of NEs + BM635 and NIs + BM859. The loaded NEs formulation is characterized by a higher microviscosity value (2.5) compared with that of NIs (0.82), probably because BM635 is more lipophilic than BM859 (8.1 and 7.31).

### 3.4. Microbiological Activity of Formulated Compounds

MIC values against *Mtb* H37Rv of free and formulated BM635 and BM859 are reported in Table 3. Although NIs + BM859 showed potent antimycobacterial activity (MIC = 0.6 µM), comparable to that of the free compound, NEs + BM635 completely lost the activity. This is most probably caused by the fact that BM635, being highly hydrophobic, encounters maximum hindrance in diffusion whereas BM859, characterized by high solubility in aqueous medium (152 µM), diffuses more easily. To date, empty NEs and NIs have not show any influence on mycobacterial growth, indicating that these formulations do not have any anti-mycobacterial activity per se [27].

## 4. Conclusions

Current tuberculosis treatment, consisting of multi-drug administration for a long period of time, is challenging because it presents drug-related side effects and poor patient compliance. The risk of patients stopping the therapy too early, and the emergence of MDR-TB is high. Nanocarriers have the advantage of mitigating drug limitations by improving stability, solubility, and bioavailability to the target site, thus reducing off-target effects. In this study, NEs and NIs nanocarriers have been used to entrap two potent anti-mycobacterial compounds. During preliminary experiments, screenings were done to select the most appropriate nanocarrier for both compounds and, even if it is well known that niosomal vesicles are able to entrap both lipophilic and hydrophilic drugs [10,11,12], the entrapment of lipophilic BM635 into NIs was negligible, whereas more hydrophilic BM859 can be successfully loaded into NIs. Due to this, the more lipophilic BM635 was loaded into different nanocarriers (NEs with high EE). Both formulations exhibited good stability in simulated biological media. Unfortunately, only NIs + BM859 showed activity against *Mtb*, whereas NEs + BM635 seemed to be completely inactive. 

These preliminary results highlighted that the choice of nanocarriers needs to be related to drug features and that the niosomal formulation described in this work could be a useful therapeutic tool for tuberculosis treatment.

The biological results need to be confirmed by further studies to understand the in vivo behavior of selected formulations.

## Figures and Tables

**Table 1:** Sample compositions.

Sample	Tween 20 (mg/mL)	Almond Oil (mg/mL)	BM635 Loaded (mg/mL)	Cholesterol (mg/mL)	BM859 Loaded (mg/mL)
NEs	18.4	18.4	-	-	-
Nes + BM635			4.6		
NIs		-	-	5.8	-
Nis + BM859					3.8

**Table 2:** Physical-chemical properties of NEs/NIs formulations.

Sample	Hydrodynamic Diameter (nm) ± SD	ζ-Potential (mV) ± SD	PDI ± SD	Drug-Entrapment Efficiency (E.E.%)	I_1_/I_3_ (Polarity)	I_E_/I_3_ (Microviscosity)
NEs	132.6 ± 3.4 159.5 ± 4.6	−21.9 ± 1.2 −25.9 ± 2.3	0.1 ± 0.01	-	0.96	1.87
NEs + BM635			0.1 ± 0.01	95.6	0.97	2.15
NIs	161.0 ± 3.3 172.5 ± 4.2	−28.5 ± 1.4 −19.4 ± 3.3	0.1 ± 0.01	-	1.26	0.67
NIs + BM859			0.1 ± 0.01	81.6	1.32	0.82

**Table 3:** Activity against *Mtb* H37RV (MIC) of NEs + BM635 and NIs + BM859 compared to free BM635 and BM859.

Compound	MIC (µM)
Nes + BM635	40
BM635	0.12
NEs	ND ^a^
Nis + BM859	0.6
BM859	0.3
NIs	ND ^a^

^a^ Not determined.

## Supplementary Materials

The following are available online at www.mdpi.com/article/10.3390/pharmaceutics14030610/s1, Table S1: Composition of all studied NEs formulations in the ternary diagram; in bold, sample composition in the red region, Figure S1: Synthetic pathway for BM635, Figure S2: Synthetic pathway for BM859, Figure S3: Physico-chemical stability up to 60 days of drug loaded into nanocarriers. Effect of storage temperature (room temperature, RT; and 4 °C) in terms of drug concentration for BM635-loaded NEs (Panel A) and for BM859-loaded NIs (Panel B), Figure S4: Stability studies in the presence of simulated intestinal fluid (SIF), simulated gastric fluid (SGF) and human serum (HS) following variation in terms of hydrodynamic diameter and ζ-potential, panel A and B for empty NEs and empty Nis, respectively.
