# Supplementary material for: Nano-Based Drug Delivery Systems of Potent MmpL3 Inhibitors for Tuberculosis Treatment
Source: Pharmaceutics. 2022 Mar 10;14(3):610. doi: 10.3390/pharmaceutics14030610 (PMC8955761; doi:10.3390/pharmaceutics14030610)
Supplement: Supplementary file 1 [file pharmaceutics-14-00610-s001.zip › pharmaceutics-1600684-supplementary.pdf]

pharmaceutics

Supporting Information

Nano-Based Drug Delivery Systems of Potent MmpL3 Inhibitors for Tuberculosis Treatment

Patrizia Nadia Hanieh, Sara Consalvi, Jacopo Forte, Gianluigi Cabiddu, Alessandro De Logu, Giovanna Poce, Federica Rinaldi, Mariangela Biava, Maria Carafa and Carlotta Marianecci

Chemistry

BM635 was prepared following a straightforward synthetic pathway reported in Figure S1. Briefly, 1,4-diketone **3** was obtained by treating the 4-isopropylbenzaldheyde **1** with methyl vinyl ketone **2** in a sealed glass tube in the microwave reactor. Microwave assisted cyclization of **3** in the presence of 4-F-aniline gave the expected pyrrole **4** in good yield. Finally, by treating pyrrole **4** with formaldehyde and morfoline, following Mannich reaction conditions, BM635 was obtained.

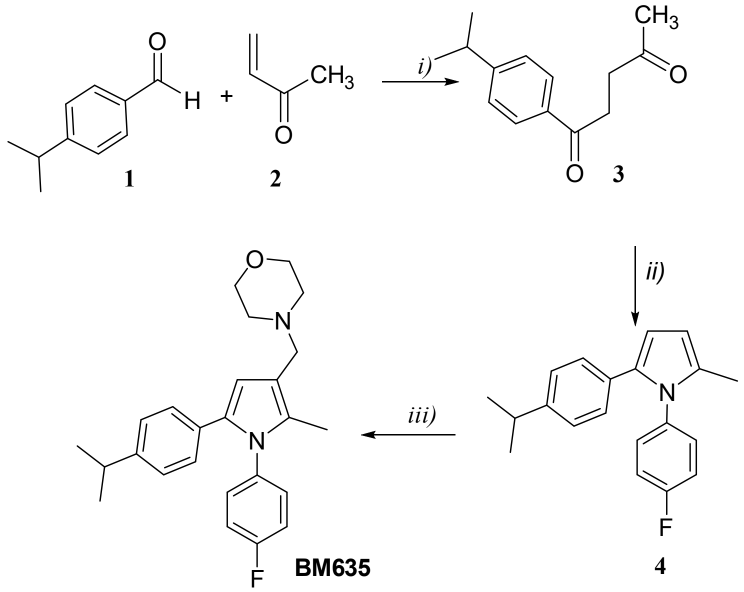

Reagents and conditions: *i)* 3-ethyl-5-(2-hydroxyethyl)-4-methylthiazolium bromide, TEA, microwave, 15 min; *ii)* 4-F-aniline, *p*-toluensulfonic acid, EtOH, microwave, 30 min; *iii)* morfoline, CH_3CN, HCHO, CH_3COOH, room temperature, 1 h.

Figure S1. Synthetic pathway for BM635.

BM859 was prepared as shown in Figure S2. The reaction between ketone **5** and diethyloxalate **6** in the presence of lithium bis(trimethylsilyl)-amide gave the desired lithium salt **7** that was in turn cyclized with 4-F-phenylhydrazine **8** to afford pyrazole **9**. Ethyl ester **9** was converted into the corresponding pyrazole-3-carbaldehydes **11** in two steps, $LiAlH_4$ reduction and Dess-Martin periodinane oxidation. Finally, **11** underwent reductive amination with 4-methoxypiperidine amine in the presence of $NaBH(CH_3COO)_3$ to produce the BM859.

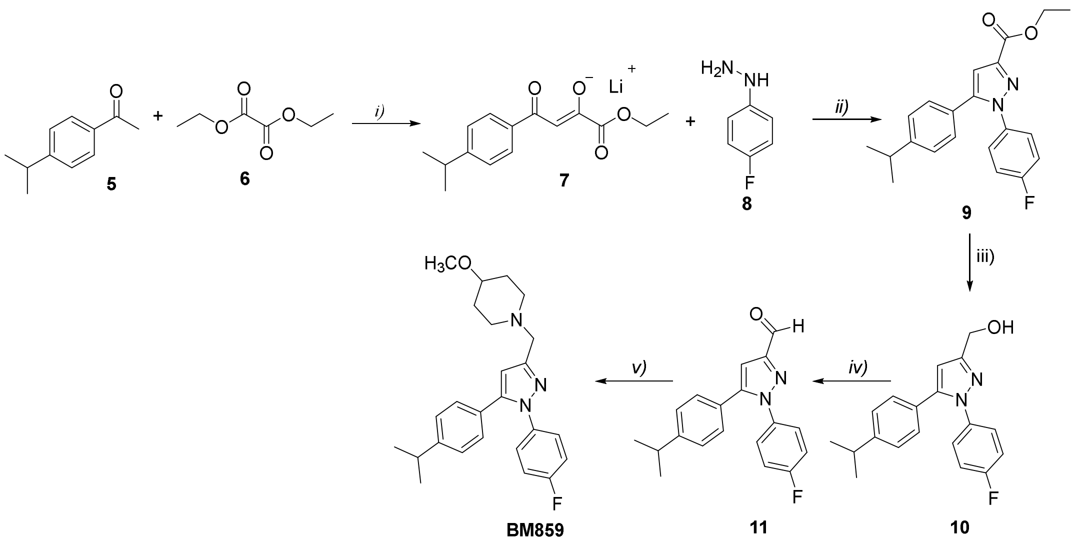

Reagents and conditions: i) $LiN(Si(CH_3)_3)_2$, THF, -78 °C and then room temperature, 24 h; ii) EtOH, 90 °C, 5h; iii) $LiAlH_4$, THF, 0 °C and then room temperature, 3h; iv) Dess-Martin periodinane, DCM, room temperature, 30 min; v) 4-methoxypiperidine, CH_3COOH, $NaBH(CH_3COO)_3$, DCE, room temperature, 2h.

Figure S2. Synthetic pathway for BM859.

Table S1. Composition of all studied NEs formulations in the pseudoternary diagram; in bold, sample composition in the red region.

	Almond oil (g)	Tween 20 (g)	Hepes Buffer (g)	Almond oil (%*w*/*w*)	Tween 20 (%*w*/*w*)	Hepes Buffer (%*w*/*w*)
1	0.25	0.50	0.25	25	50	25
**2**	**0.17**	**0.33**	**0.50**	**17**	**33**	**50**
**3**	**0.12**	**0.25**	**0.63**	**12**	**25**	**63**
**4**	**0.07**	**0.15**	**0.78**	**7**	**15**	**78**
**5**	**0.03**	**0.06**	**0.91**	**3**	**6**	**91**
**6**	**0.02**	**0.04**	**0.94**	**2**	**4**	**94**
**7**	**0.01**	**0.02**	**0.97**	**1**	**2**	**97**
**8**	**0.30**	**0.20**	**0.50**	**30**	**20**	**50**
**9**	**0.50**	**0.10**	**0.40**	**50**	**10**	**40**
**10**	**0.60**	**0.05**	**0.35**	**60**	**5**	**35**
11	0.10	0.50	0.40	10	50	40
12	0.70	0.10	0.20	70	10	20
13	0.80	0.10	0.10	80	10	10
14	0.40	0.40	0.20	40	40	20
15	0.10	0.70	0.20	10	70	20
**16**	**0.30**	**0.30**	**0.40**	**30**	**30**	**40**
17	0.50	0.30	0.20	50	30	20
18	0.30	0.40	0.30	30	40	30
19	0.02	0.70	0.28	2	70	28

20	0.80	0.05	0.15	80	5	15
21	0.15	0.55	0.30	15	55	30
22	0.45	0.25	0.30	45	25	30
**23**	**0.20**	**0.40**	**0.40**	**20**	**40**	**40**
**24**	**0.40**	**0.20**	**0.40**	**40**	**20**	**40**
**25**	**0.40**	**0.10**	**0.50**	**40**	**10**	**50**
26	0.60	0.30	0.10	60	30	10
27	0.45	0.45	0.10	45	45	10
28	0.30	0.60	0.10	30	60	10
29	0.25	0.70	0.5	25	70	5
30	0.60	0.20	0.20	60	20	20
31	0.72	0.13	0.15	72	13	15
**32**	**0.30**	**0.10**	**0.60**	**30**	**10**	**60**
33	0.70	0.05	0.25	70	5	25
34	0.50	0.20	0.30	50	20	30
35	0.60	0.10	0.30	60	10	30
36	0.35	0.30	0.35	35	30	35
37	0.20	0.45	0.35	20	45	35
**38**	**0.05**	**0.45**	**0.50**	**5**	**45**	**50**
**39**	**0.20**	**0.15**	**0.65**	**20**	**15**	**65**
**40**	**0.25**	**0.05**	**0.70**	**25**	**5**	**70**
41	0.58	0.17	0.25	58	17	25
42	0.50	0.15	0.35	50	15	35
**43**	**0.66**	**0.02**	**0.32**	**66**	**2**	**32**
44	0.72	0.01	0.27	72	1	27
45	0.35	0.35	0.30	35	35	30
46	0.05	0.50	0.45	5	50	45
47	0.02	0.57	0.41	2	57	41
48	0.08	0.57	0.35	8	57	35
**49**	**0.02**	**0.48**	**0.50**	**2**	**48**	**50**
50	0.30	0.37	0.33	30	37	33
51	0.30	0.33	0.37	30	33	37
**52**	**0.02**	**0.02**	**0.96**	**2**	**2**	**96**

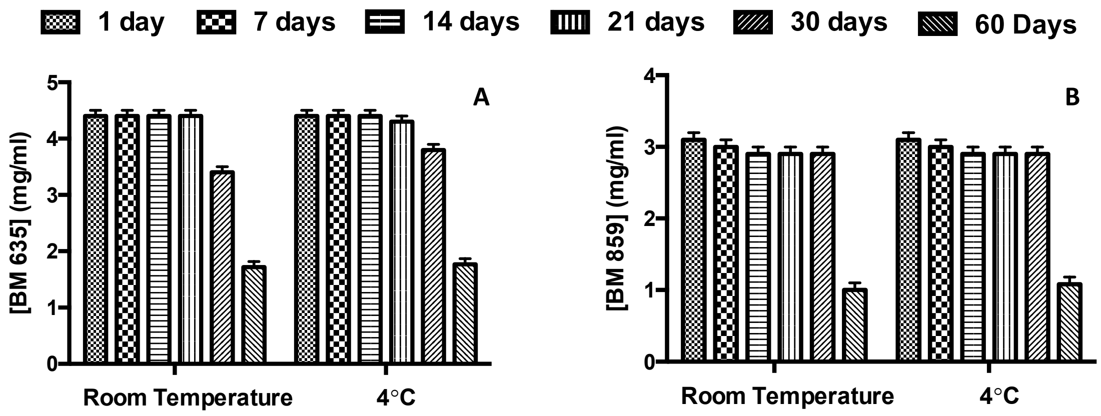

Figure S3. Physico-chemical stability up to 60 days of drug loaded into nanocarriers. Effect of storage temperature (room temperature, RT; and 4 °C) in terms of drug concentration for BM635-loaded NEs (Panel A) and for BM859-loaded NIs (Panel B).

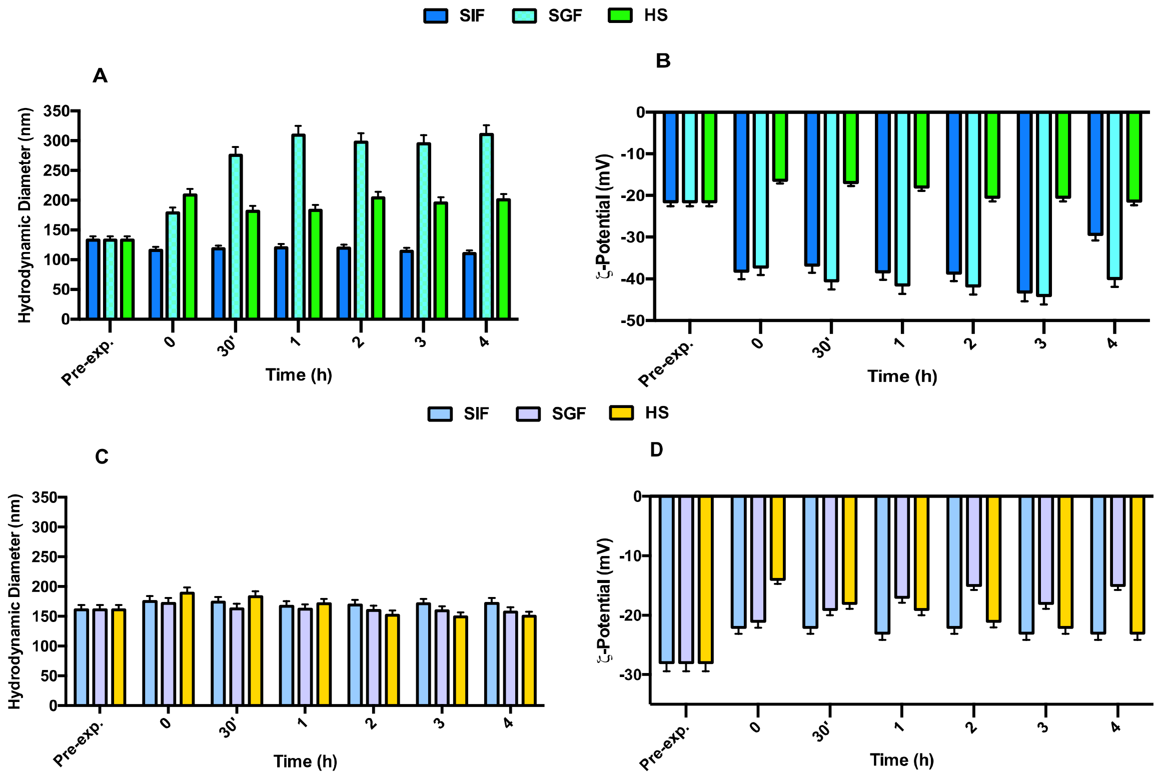

Figure S4. Stability studies in presence of simulated intestinal fluid (SIF), simulated gastric fluid (SGF) and Human serum (HS) following variation in terms of hydrodynamic diameter and ζ-potential, panel A and B for empty NEs and empty NIs respectively. Reported data represent the mean of three experiments ±SD.
